# The Impacts of Globalization on Inequality in the Post-COVID-19 World: Evidence From China

**DOI:** 10.3389/fpubh.2021.790312

**Published:** 2021-11-29

**Authors:** Tsun Se Cheong, Yanrui Wu, Michal Wojewodzki, Ning Ma

**Affiliations:** ^1^Department of Economics and Finance, The Hang Seng University of Hong Kong, Hong Kong SAR, China; ^2^Department of Economics, The University of Western Australia, Perth, WA, Australia; ^3^School of Financial Management, Hainan College of Economics and Business, Hainan, China

**Keywords:** county level, globalization, inequality, COVID-19, China, C5, F6, R11

## Abstract

Empirical studies suggest that globalization (FDI and international trade) has been greatly affected by the COVID-19 and related anti-pandemic measures imposed by governments worldwide. This paper investigates the impact of globalization on intra-provincial income inequality in China and the data is based on the county level. The findings reveal that FDI is negatively associated with intra-provincial inequality, intra-provincial inequality increases as the primary industry sector (agriculture) declines. The result also finds that the increase in inequality stems not from the development in the tertiary or secondary industry sectors per se, but the unevenness in the distribution of these sectors.

## Introduction

Since opening and economic reforms in 1978 Chinese GDP (GDP per capita) experienced a period of unprecedented growth, increasing from 367.9 billion Yuan (385 Yuan) in 1978 to over 90 trillion Yuan (64,644 Yuan) in 2018^1^. During the same period, over 850 million Chinese were lifted out of poverty (1). Many attribute this impressive achievement to the successful implementation of globalization in the reforms [e.g., (2–6)].

However, income inequality in China escalated in unison with the reduction in aggregate poverty levels. Thus, Chinese policymakers tried to combat income inequality. Against this backdrop, after 7 years of a moderate decline (from 2008 to 2015), income inequality has been on the rise (7). The increase in inequality at different spatial levels questions the benefits of globalization. Therefore, it is of interest to investigate if globalization is responsible for the increase in intra-provincial (county-level) inequality in China^2^.

According to the Asian Development Bank (8), spatial inequality accounted for 54% of China's income inequality in 2007. Cheong and Wu (9) show that intra-provincial regional inequality in China grew substantially between 1997 and 2007. Regrettably, most of the studies on China's income inequality focus on national [e.g., (10, 11)] or inter-provincial level [e.g., (12, 13)]. To the best of our knowledge, there is no study examining the association between globalization and county-level intra-provincial regional inequalities in Chinese provinces. Thus, the above studies and their findings remain impractical for the Chinese policymakers tackling regional inequality amongst counties and county-level cities within each province.

This paper contributes to the literature by studying the impacts of globalization on intra-provincial regional inequality in China from 1997 to 2007. Our findings might help to derive pragmatic and efficient policies mitigating regional inequality within Chinese provinces. Moreover, the results contribute to the ongoing scholarly debate [e.g., (13–15)] as to whether the link between globalization and inequality is justified. Our findings suggest that FDI negatively affects intra-provincial inequality. Furthermore, we find that industrialization and development of the secondary industry sector (manufacturing and construction) and the tertiary industry sector (services) are positively associated with regional income inequality in China. Moreover, the results suggest that the transportation infrastructure decreases intra-provincial inequality.

Empirical (16–18) and anecdotal evidence suggests that globalization (FDI and international trade) has been greatly affected by the COVID-19 and related anti-pandemic measures (e.g., quarantines, lockdowns, and social distancing) imposed by the governments worldwide. Furthermore, COVID-19 in conjunction with the ongoing US-Sino trade war poses an unprecedented threat to China's social and economic prosperity. In particular, high-income inequality in pre-pandemic China documented at various spatial levels (9, 19, 20) might become more acute in the short- and long-run post-pandemic perspective. For instance, COVID-19 related closures and lockdowns have a larger knock-on effect on jobs and incomes of the lower-income, less-educated households from poor regions who are less likely to work remotely and unable to relocate to the rich regions (21–24). On the other hand, lower-income people who were allowed to commute to work were more likely to be infected with COVID-19 while less likely to receive adequate healthcare provision than high-income patients (25). On top of that, unlike many governments in developed economies (e.g., the U.S., the U.K., Germany), Chinese governments did not offer cash support to the affected vulnerable households.

While the above-listed factors can be considered as mainly short-term, COVID-19 might also affect the labor market and income disparity in the long-term due to a persistent increase in the online and teleworking sector requiring IT skills and access to technology. This, in turn, works to the advantage of a better-educated labor force from richer regions [e.g., (26)]. Moreover, social distancing measures brought prolonged closures of schools and distance/online learning. Such arrangements are expected to have a disproportionately large negative effect on the education of pupils from low-income families, and thus their future employment opportunities (27). Summing up, empirical and anecdotal evidence suggests that COVID-19 hampered globalization but increased poverty and income inequality globally and in China. While it is still too early for empirical research examining the long-lasting effect of COVID-19 on the association between globalization and regional income inequality in China, our study documents such association in earlier years and thus constitutes an important reference for the post-COVID-19 studies.

The remainder of this paper is structured as follows. Section Literature Review presents a review of the literature. Section Methodology delivers the discussions of the econometric model and introduces the data. Section Results and Discussions presents the results and interpretations, while Section Robustness Tests delivers and discusses robustness tests. Section Conclusions concludes the paper and offers policy recommendations.

## Literature Review

Foreign direct investment (FDI) and international trade are the most common proxies of globalization. According to NBSC (7), inward FDI in China increased enormously from US$1.96 billion in 1985 to US$135 billion in 2018. The positive impact of FDI on economic growth can be linked with the efficiency spillovers to domestic firms [e.g., (28, 29)]. Furthermore, FDI can bring in new products and managerial technologies as well as advanced organizational arrangements. Many cross-country studies suggest that FDI boosts economic growth [e.g., (30, 31)]. Focusing on China, Tian et al. (5) find that the provinces with a high FDI ratio experience faster technology updating and higher economic growth. In tandem with inward FDI, Chinese international trade surged from US$20.6 billion in 1978 to US$4.62 trillion in 2018 (7). Numerous studies show that international trade exerts a positive impact on economic growth in China [e.g., (2–4, 6)].

Against this backdrop, the Chinese government established preferential policies to boost FDI and international trade. For instance, special-economic zones (e.g., Shenzhen), open cities, preferential exchange rates, and taxes all aim to attract foreign investors and promote international trade (32). However, it is argued that globalization could be harmful in developing countries [see (33, 34)]. Based on a sample of 1,254 empirical results from 123 peer-reviewed studies, Heimberger (15) documents a moderately positive effect of globalization on inequality in developing countries.

With regards to China, many studies report that FDI spurs inequality (35–37). Furthermore, FDI can exert different impacts on growth across regions. Tian et al. (38) document that the productivity of Chinese firms in the peripheral inland region is adversely affected by the FDI flowing into coastal regions. They argue that FDI is positively associated with regional inequality in China. Huang and Wei (19) and Zhang et al. (20) find that FDI contributes to income inequality and the formation of convergence clubs across prefectural-level Chinese cities. On the contrary, Ma and Jia (39) document a positive association between FDI and regional income convergence.

Another major component of globalization: international trade is reported by some as a major force behind the growing disparity between the inland and coastal regions [e.g., (40, 41)]. Furthermore, Zhang and Zhang (35), Gries and Redlin (42), and Wang and Chen (43) find that regional inequality in China increases with exposure to international trade. However, in a study on international trade and rural-urban inequality in Chinese prefectural-level cities, Wei and Wu (44) document that trade openness significantly reduces inequality. Additionally, some researchers report no effect of international trade on regional inequality [e.g., (13, 45)].

In summary, the common consensus is that globalization brought about by FDI can increase the output of a region. However, the effect is bi-directional. On the one hand, inequality is reduced if FDI is accurately directed at the poor and underdeveloped regions. On the other hand, inequality increases if globalization further reinforces the economic growth of the already globalized regions. Moreover, both facets of globalization (FDI and international trade) are found to exert different impacts on economic growth across the regions. This, in turn, exacerbates regional inequality in China. Importantly, most of the studies on China use provincial-level data, while the impacts of globalization on intra-provincial regional inequality remain largely unexplored by the academic community^3^.

## Methodology

### Econometric Specification

To examine the determinants of regional income inequality at the county-level in China, the regression approach is used with a baseline model presented in equation (1) below.


(1) $$\begin{array}{l} {GINI}_{i,t}=\beta_{k}X_{i,t}+\varphi_{i}+v_{t}+\varepsilon_{i,t} \end{array}$$

where *GINI*_*i, t*_ is the Gini coefficient for province *i* at time *t*, *X*_*i, t*_ is the matrix for the provincial characteristics, β_*k*_ is the *k* x 1 vector of the coefficients on *X*_*i, t*_, φ_*i*_ represents the fixed effects for province *i*, *v*_*t*_ is the set of time dummy variables. ε_*i, t*_ is the idiosyncratic disturbance term, uncorrelated across the provinces.

The results presented in this study are based on the generalized method of moments (GMM) estimator developed by Arellano and Bond (46) and Blundell and Bond (47). GMM is recognized as successful in handling endogeneity [e.g., (48, 49)]. The endogeneity is especially common in studies on income inequality, as the problems of simultaneity, unobserved heterogeneity, reverse causality, and omitted variables may all contribute to it (13, 50). One example is government expenditure supporting underdeveloped regions. It can be expected that higher expenditure will lead to a reduction in income inequality (the dependent variable). However, higher inequality may also lead to higher expenditure (the explanatory variable).

GMM estimator can overcome the endogeneity problem, control for fixed effects and time effects. Furthermore, it is recommended for unbalanced panel data with multiple endogenous variables (48, 51). Thus, the GMM estimator is often employed in the recent empirical literature on income inequality [e.g., (13, 45, 52)]. We employ the two-step system GMM (2S-SGMM) estimator by Blundell and Bond (47) which is asymptotically efficient and robust to any pattern of cross-correlation and heteroskedasticity (52).

Furthermore, to mitigate the problem of potentially downward-biased standard errors, in all specifications, we use small-sample corrected standard errors (53). Besides, because the panel dataset is unbalanced, the transformation of orthogonal deviations is used to minimize the number of gaps in the transformed equations (52). Moreover, all explanatory variables are treated as endogenous. To combat the problem of instrument proliferation (51, 52), the instruments are combined into smaller sets by collapsing blocks in the instrument matrix. Additionally, we use “*collapsed*” instruments.

### Data Sources and Explanations of Variables

Intra-provincial regional inequality amongst the counties and county-level cities (measured by the Gini coefficient) is the dependent variable. The Gini coefficient ranges from zero to 100, with zero (100) representing perfect income equality (inequality). In the baseline model (**Table 2** in Section Results and Discussions), we employ a set of 11 explanatory variables based on provincial characteristics^4^. All the provinces and autonomous regions in China are included in this study^5^. The dataset is an unbalanced panel covering the 1997–2007 period and the dataset is from Cheong and Wu (50). There is no previous research focused on the intra-provincial data and it is the first paper to apply the intra-provincial data to investigate the relationship between globalization and inequality. In addition, China's international trade was booming during that period and it is worth examining the relationship between globalization and inequality for that period. Explanatory variables are compiled from *China Statistical Yearbook* and augmented with data from *China Statistical Yearbook for Regional Economy* and *Provincial Yearbook* (7). When appropriate, data series have been adjusted for inflation by converting them to 1997 constant prices using provincial consumer price index (CPI) as the deflator.

Based on prior empirical research [e.g., (37, 43)] we use the *FDI* and a combined value of exports and imports (*XIM*) as explanatory variables proxying for the effects of globalization on intra-provincial inequality. Both variables are expressed as a share of provincial gross regional product (GRP). Furthermore, we control for the potential effect of domestic trade (*DOMTR*) measured as the ratio of retail sales to provincial GRP.

The share of the tertiary (secondary) industry outputs in Chinese national GDP changed from 43.0 (46.9%) in 2010 to 52.2% (40.7%) in 2018 (7). This means that especially the development of the tertiary industry sector has been fast in recent years^6^, which, in turn, may affect regional income inequality. Some studies find that Chinese industrialization led to a surge in inequality (40, 54) and the formation of income convergence clubs (20). Therefore, we control for the potential effects of the secondary (*SECIND*) and tertiary (*TERIND*) industry sectors on income inequality.

GDP and GRP per capita are routinely employed as a determinant of income inequality in cross-country [e.g., (14, 55, 56)] and China-focused studies [e.g., (10, 13)]. Thus, we include the real GRP per capita (*GRPPC*)^7^ explanatory variable in the model of regional inequality.

Chen and Groenewold (57) and Fan et al. (58) document a positive impact of transportation infrastructure on regional development in China, while Gries and Redlin (42) find that transportation aggravates inequality. However, the above studies are based on provincial-level data. We examine the impact of transportation infrastructure (*TRANSP*) on intra-provincial inequality^8^. Lau (6) shows that high inflation is detrimental to economic growth, while many studies suggest that there is a positive association between income inequality and inflation [e.g., (13, 60)]. Following prior studies, we include the *INLF* term in our models.

Wan (61) states that the effects of fiscal transfers on inequality are generally negligible and sometimes negative at the provincial level. Fang and Rizzo (62) claim that government transfers are ineffective in inequality reduction in rural China. On the contrary, Zhuang and Li (63) and Jain-Chandra et al. (10) argue for an important role of fiscal transfers in reducing inequality. However, the association between government expenditure and intra-provincial inequality has not been studied. Given the above, we employ the *GOVEX* term calculated as a ratio of government expenditure supporting underdeveloped areas to provincial GRP. Another variable (*ECOSIG*) is the share of provincial GRP to national GDP. We use the *ECOSIG* term to test the association between the relative provincial output and intra-provincial inequality.

Shindo (64) concludes that education subsidies in the eastern, coastal province of Liaoning and Jiangsu boost economic growth and welfare. However, he also argues that due to “large differences in productivity between the regions, the growth gap widens with evenly raised education subsidy rates.” (p. 1061). Prior studies suggest that the income disparity can be largely explained by educational levels [e.g., (10, 65)]. We investigate the impact of education on intra-provincial inequality by including the ratio of educational funding in each province to provincial GRP (*EDUFND*) in our baseline model.

### Descriptive Statistics

**Figure 1:**
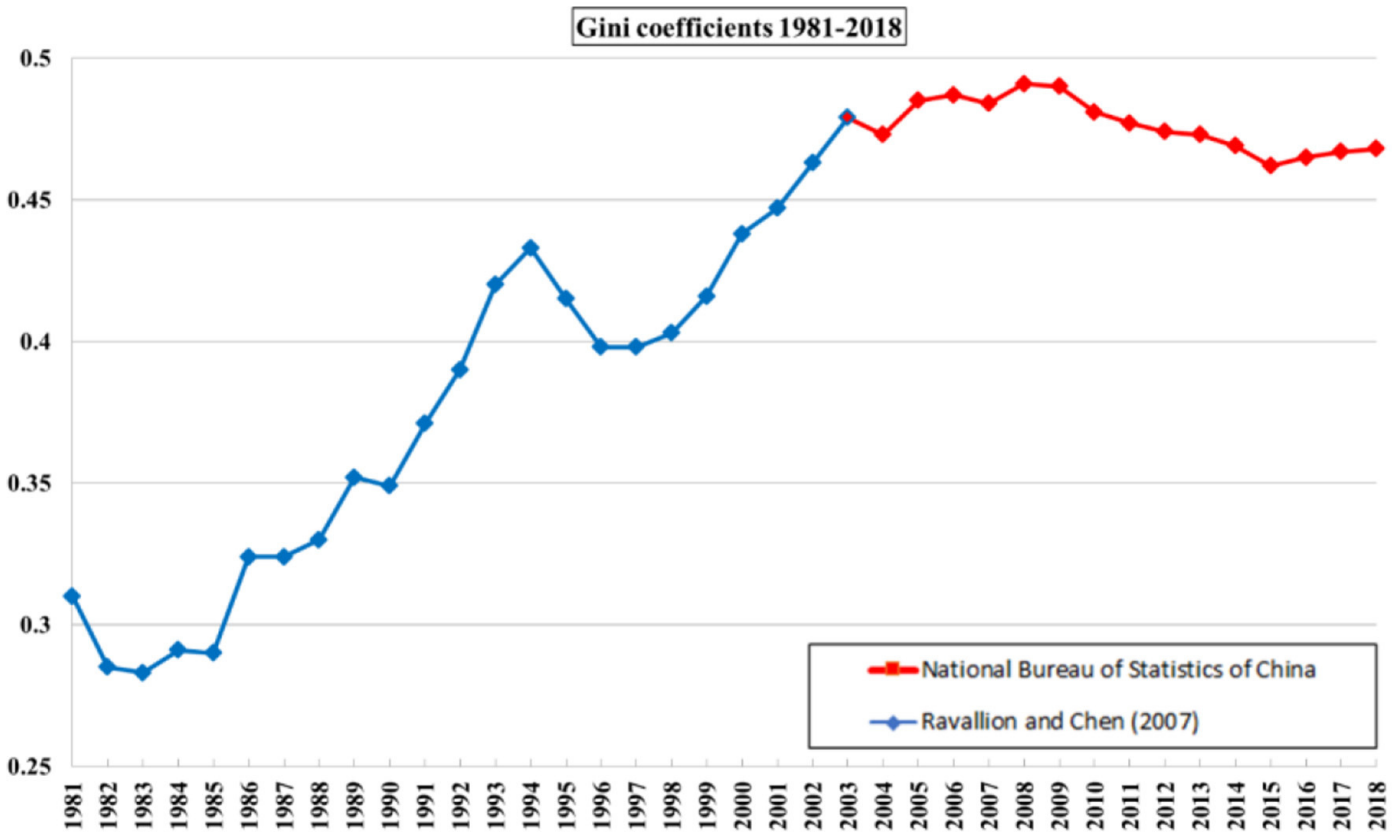
Income inequality in China from 1981 to 2018 measured by Gini coefficients. Source: Ravallion and Chen (32) and NBSC (7).

Figure 1 presents the plot of Gini coefficients for China from 1981 to 2018. Overall, inequality has been on the rise, except for three periods of decline: 1981–1983, 1994–1996, and 2008–2015. Furthermore, in 2008 a Gini coefficient peaked by reaching a value of 0.491. Figure 1 also indicates that income inequality increased moderately from 0.462 to 0.468 during the most recent 3 years. Moreover, comparing the Gini coefficient for 1981 and 2018, it has grown by over 50%. Overall, Figure 1 questions the effectiveness of policymakers' efforts to combat China's high and persistent inequalities.

Looking at Table 1, we can observe that the regional inequality for Chinese provinces between 1997 and 2007 ranges from 0.141 to 0.469, while the dispersion of *GINI* (coefficient of variation CV) equals 0.254. This means that the standard deviation accounted for around 25% of the *GINI's* mean value. Summing up, there are substantial disparities in the sample's intra-provincial income inequality.

**Table 1:** Descriptive statistics.

**Variable**	**Obs**.	**Mean**	**Std. Dev**.	**CV**	**Min**	**Max**
*GINI*	279	0.283	0.072	0.254	0.141	0.469
*FDI*	294	0.013	0.012	0.923	0.000	0.062
*XIM*	297	0.240	0.320	1.333	0.040	1.875
*DOMTR*	295	0.342	0.044	0.129	0.219	0.455
*SECIND*	297	0.450	0.081	0.180	0.198	0.600
*TERIND*	297	0.369	0.044	0.119	0.254	0.556
*GRPPC*	297	9.552	5.566	0.583	2.215	33.681
*TRANSP*	297	0.084	0.047	0.560	0.013	0.257
*INLF*	295	101.358	2.149	0.021	96.800	106.644
*GOVEX*	270	0.003	0.004	1.333	0.000	0.017
*ECOSIG*	297	0.033	0.027	0.818	0.001	0.114
*EDUFND*	297	0.046	0.015	0.326	0.25	0.123

## Results and Discussions

**Figure 2:**
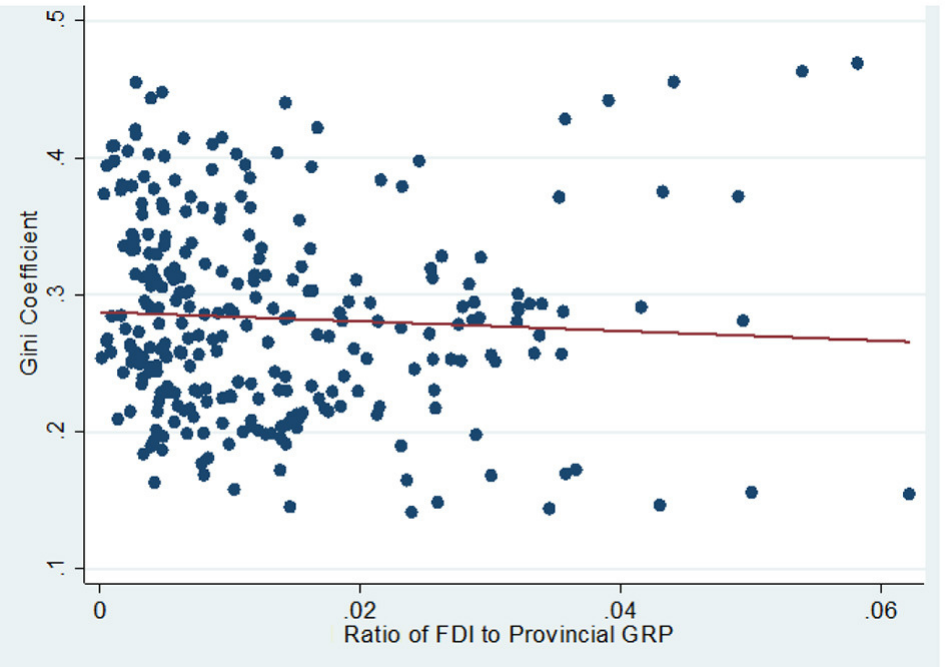
Intra-provincial regional inequality (GINI variable) and the ratio of FDI to provincial GRP (FDI variable).

**Figure 3:**
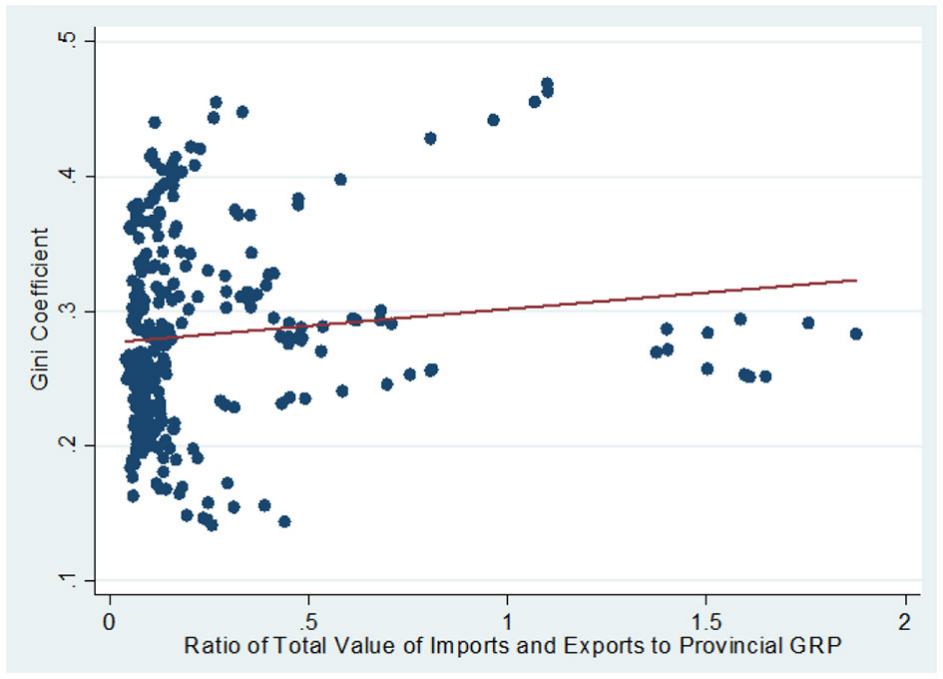
Intra-provincial regional inequality (GINI variable) and the ratio of the total value of imports and exports to provincial GRP (EXIM variable).

The correlation between two measures of globalization (FDI and international trade) and intra-provincial regional inequality can be observed in Figures 2, 3. The scatterplot and the line of best fit shown in Figure 2 (3), suggest that regional inequality is somewhat negatively (positively) correlated with FDI (international trade). These observations suggest that although FDI and international trade are major components of globalization, they have potentially different effects on Chinese intra-provincial regional inequality.

Empirical results corresponding to equation (1) shown in Table 2 are based on the 2S-SGMM estimator^9^^,^^10^. Sargan and Hansen tests suggest that the instruments are valid, while the AR (2) test indicates the absence of the second-order autocorrelation in differenced residuals Δε_*i, t*_ in the transformed equation, i.e., the 2S-SGMM estimator is correctly specified.

**Table 2:** Regional inequality and globalization.

**Independent variable**	
*FDI*	−3.526**
	(1.497)
*XIM*	0.030
	(0.033)
*DOMTR*	−0.393**
	(0.166)
*SECIND*	0.708**
	(0.269)
*TERIND*	1.020*
	(0.503)
*GRPPC*	0.011*
	(0.006)
*ECOSIG*	−1.028
	(2.035)
*TRANSP*	0.574**
	(0.244)
*INLF*	−0.036**
	(0.014)
*GOVEX*	−0.076
	(4.538)
*EDUFND*	0.808
	(2.147)
Time dummies	Yes
Provincial dummies	Yes
Observations:	251
Period of estimation	1997–2007
AR(2) test (*p*-value)	0.107
Sargan test (*p*-value)	0.285
Hansen test (*p*-value)	0.798

The dependent variable is intra-provincial regional inequality (GINI) measured by the Gini coefficient in each province. Standard errors (in parentheses) are asymptotically robust to heteroskedasticity. AR(2) is the Arellano-Bond test for the second-order serial correlation in the first-differenced residuals, under the null hypothesis of no serial correlation. Both Sargan and Hansen are tests of the overidentifying restrictions under the null hypothesis of valid instruments. *, **, and *** show that estimated coefficients are statistically significant at the 10, 5, and 1% levels. See Table A1 in the Appendix for a list of all variables and their definitions. In all specifications, we employ untabulated year and provincial dummy variables.

The estimated coefficient on *FDI* (-3.526) is statistically significant at the 5% level and negative which indicates that FDI can alleviate intra-provincial inequality. Given the sample average value of *FDI* (0.013) and *GINI* variable (0.283), we can expect that a 10% increase in FDI to provincial GRP ratio brings about a 0.016 decrease in intra-provincial inequality (measured by *GINI* coefficient), ceteris paribus^11^. The result corroborates the findings of Gries and Redlin (42), and Ma and Jia (39). However, our finding differs from studies based on inter-provincial data [e.g., (35, 37)] and prefectural-level cities data (19) documenting that FDI increases inequality.

Mixed findings at different spatial levels are reported in studies on international trade. Many studies based on provincial-level data show that trade increases inequality [e.g., (35, 40, 43)]. However, Wei and Wu (44) reach the opposite conclusion using prefectural- and municipality-level data. Hence, our results pinpoint that policy formulation at the county-level should not be based on research carried out at the provincial- or prefectural-level, and vice versa. Furthermore, research on regional inequality should be carried out at various spatial levels.

Table 2 shows that the proportion of retail sales of consumer goods to provincial GRP (*DOMTR*) is negatively associated with regional inequality. The *DOMTR* variable refers to the sum of retail sales of commodities sold by wholesale and retail trades, catering services, publishing, post and telecommunications, and other services to urban and rural households' consumption and social institutions' public consumption. It might be, that as most of these services do not require highly skilled labor, the expansion of these sectors can absorb the surplus of unskilled labor. Consequently, the backward regions within a province can enter these services easily, which, in turn, improves the living standards of poor regions. In other words, our findings suggest that the promotion of domestic trade in the underdeveloped regions can increase their outputs, and thus alleviate intra-provincial inequality.

The statistically significant and positive estimated coefficient on *SECIND* and *TERIND* terms imply that inequality increases with the decline of agriculture (primary industry sector). Given the sample mean value of *SECIND* (0.450) and *TERIND* (0.369), we can expect that a 1% increase in a share of the secondary (tertiary) sector in provincial GRP brings is associated with a rise of 0.011 (0.013) in regional inequality, holding all else constant. This finding agrees with other studies [e.g., (32, 57, 62, 65)]. The returns of the secondary and tertiary industry sectors are higher than that of the primary industry sector. Therefore, coupled with the documented uneven distribution of industrialization and development across the regions, an expansion of these sectors can increase intra-provincial regional inequality.

Table 2 shows that the estimated coefficient of *GRPPC* is statistically significant and positive, which is in line with e.g., Xiong (13). This implies that the inequality levels in rich provinces are higher than those in poor provinces. Our results suggest that, when governments formulate a policy for inequality alleviation, they should focus on the poor and the rich provinces alike. This also calls for the design of a comprehensive intra-provincial map of China and a coherent strategy combating inequality in all the provinces.

The estimated coefficient on the *TRANSP* variable is positive and statistically significant at the 5% level. This is consistent with e.g., Gries and Redlin (42). The regions with agglomerated industries tend to be richer and have higher tax revenues, thus can finance new infrastructure, which, in turn, attracts more industries. This circularity can lead to a further increase in regional inequality. Therefore, our findings are important and call for a concerted strategy of equalizing access for all the regions. In other words, the governments should provide better access for the poor regions by improving their transportation infrastructure.

It is often suggested that income inequality and inflation are positively associated (13, 60). Contrary to these expectations, Table 2 shows that inflation is negatively associated with regional inequality. One reason we can think is that inflation has a much greater impact on regions relying on secondary and tertiary industries than those relying on primary industries. Thus, the population in the urban (industrialized) areas might be experiencing larger reductions in their real incomes due to an increase in e.g., food prices. Therefore, richer county-level units with a high level of urbanization, become poorer in real terms, while the county-level units with a low level of urbanization (agrarian-oriented) are less affected and remain similarly low levels of income. Hence, inflation's equalizing side-effect on intra-provincial income distribution.

## Robustness Tests

We test if the results are robust to different proxies and specifications. OECD (67) suggests the use of exports only, without the value of imports, in assessing the degree of globalization. Therefore, we use the proportion of the total value of exports to provincial GRP (*EX* variable). Besides, because *GRPPC* and *ECOSIG* variables are similar in their construct, we check if the results remain robust if one of them is removed from the model. Consequently, column (1) and column (2) in Table 3 lacks the *GRPPC* and the *ECOSIG* term, respectively. Moreover, Column (3) shows the results from the specification with both *GRPPC* and *ECOSIG* terms.

**Table 3:** Robustness tests for regional inequality and globalization.

**Independent variable**	**(1)**	**(2)**	**(3)**
*FDI*	−2.001**	−3.117**	−3.513**
	(0.891)	(1.509)	(1.306)
*EX*	0.242*	0.085	0.016
	(0.137)	(0.221)	(0.058)
*DOMTR*	−0.424	−0.405	−0.461***
	(0.346)	(0.337)	(0.149)
*SECIND*	0.906**	0.673*	0.702**
	(0.359)	(0.352)	(0.277)
*TERIND*	0.680*	0.608*	0.995**
	(0.375)	(0.306)	(0.456)
*GRPPC*		0.008	0.012**
		(0.008)	(0.006)
*ECOSIG*	−3.120		−1.238
	(2.175)		(1.597)
*TRANSP*	0.618**	0.762**	0.620**
	(0.250)	(0.356)	(0.245)
*INLF*	−0.023*	−0.018	−0.034***
	(0.013)	(0.014)	(0.012)
*GOVEX*	1.399	5.467	−0.338
	(5.540)	(5.060)	(4.823)
*EDUFND*	−1.703	0.230	0.672
	(1.308)	(1.727)	(1.788)
Time dummies	Yes	Yes	Yes
Provincial dummies	Yes	Yes	Yes
Observations:	251	251	251
Period of estimation	1997–2007	1997–2007	1997–2007
AR(2) test (*p*-value)	0.127	0.127	0.111
Sargan test (*p*-value)	0.058	0.277	0.349
Hansen test (*p*-value)	0.750	0.705	0.799

The dependent variable is intra-provincial regional inequality (GINI) measured by the Gini coefficient in each province. Standard errors (in parentheses) are asymptotically robust to heteroskedasticity. AR(2) is the Arellano-Bond test for the second-order serial correlation in the first-differenced residuals, under the null hypothesis of no serial correlation. Both Sargan and Hansen are tests of the overidentifying restrictions under the null hypothesis of valid instruments. *, **, and *** show that estimated coefficients are statistically significant at the 10%, 5%, and 1% levels. See Appendix Table A1 for a list of all variables and their definitions. In all specifications, we employ untabulated year and provincial dummy variables.

Column (1) in Table 3 shows that the coefficient on the *EX* variable (unlike on *EXIM* term in Table 2) is statistically significant and has a positive sign. Furthermore, coefficients on *SECIND* and *TERIND* (*FDI* and *INFL*) terms are statistically significant, positive (negative), and carry similar magnitudes as their equivalents in Table 2. Column (2) and (3) shows that the coefficients on *GRPPC* (*DOMTR*) gain statistical significance at the 5% level and carries a positive (negative) sign. In summary, the robustness tests show that the variables of *FDI, SECIND, TERIND*, and *TRANSP* (*INLF*) retain statistical significance in all (two out of three) specifications. Therefore, we can conclude that overall, the results are robust.

The *XIM* term from models in Tables 2, 3 is based on the proportion of provincial GRP. In Table 4, we test whether the results are robust to the effects of international trade based on a per capita basis: the total value of exports and imports per capita (*EXIMPC*) and exports per capita (*EXPC*). To test the robustness of the effects of education on intra-provincial inequality we use two alternative proxies for education: the number of secondary school enrolments (*EDUENR*), and secondary school graduates (*EDUGRA*) as a share of the provincial population. The estimated results are shown in Table 4 which shows that all three proxies of education (*EDUFND, EDUENR*, and *EDUGRA*) are statistically insignificant. Therefore, there is not enough evidence that education plays a major role in intra-provincial inequality in China. The coefficients on *EXIMPC* and *EXPC* are also insignificant. However, the coefficients on *FDI, SECIND*, and *TRANSP* (*TERIND, GRPPC*, and *INFL*) retain their significance, direction, and have a consistent magnitude in all (two out of three) specifications.

**Table 4:** Robustness test (ii) for regional inequality and globalization.

**Independent variable**	**(4)**	**(5)**	**(6)**
*FDI*	−3.261*	−3.297**	−3.274**
	(1.689)	(1.535)	(1.543)
*EXPC*	−0.0005	−0.001	
	(0.003)	(0.003)	
*EXIMPC*			−0.001
			(0.002)
*DOMTR*	−0.237	−0.187	−0.181
	(0.227)	(0.271)	(0.276)
*SECIND*	1.025*	0.996*	0.972*
	(0.547)	(0.519)	(0.539)
*TERIND*	1.049	1.053*	1.052*
	(0.699)	(0.582)	(0.603)
*GRPPC*	0.007	0.010**	0.009**
	(0.005)	(0.004)	(0.004)
*ECOSIG*	−0.783	−0.801	−0.532
	(1.435)	(1.302)	(1.316)
*TRANSP*	0.519*	0.698**	0.664**
	(0.298)	(0.310)	(0.281)
*INLF*	−0.027	−0.027*	−0.026*
	(0.017)	(0.014)	(0.014)
*GOVEX*	−3.692	2.497	2.735
	(9.096)	(6.656)	(7.299)
*EDUFND*	1.151	0.736	0.936
	(1.590)	(1.175)	(1.167)
*EDUENR*	−3.122		
	(2.484)		
*EDUGRA*		−4.820	−4.661
		(7.634)	(7.649)
Time dummies	Yes	Yes	Yes
Provincial dummies	Yes	Yes	Yes
Observations:	251	251	251
Period of estimation	1997–2007	1997–2007	1997–2007
AR (2) test (*p*-value)	0.124	0.104	0.116
Sargan test (*p*-value)	0.101	0.153	0.112
Hansen test (*p*-value)	0.793	0.792	0.788

The dependent variable is intra-provincial regional inequality (GINI) measured by the Gini coefficient in each province. Standard errors (in parentheses) are asymptotically robust to heteroskedasticity. AR (2) is the Arellano-Bond test for the second-order serial correlation in the first-differenced residuals, under the null hypothesis of no serial correlation. Both Sargan and Hansen are tests of the overidentifying restrictions under the null hypothesis of valid instruments. *, **, and *** show that estimated coefficients are statistically significant at the 10%, 5%, and 1% levels. See Appendix Table A1 for a list of all variables and their definitions. In all specifications, we employ untabulated year and provincial dummy variables.

## Conclusions

This paper investigates the impact of globalization on intra-provincial income inequality in China during the 1997–2007 period. Unlike most prior studies based on inter-provincial data, our findings are based on county-level data. Therefore, this study pinpoints the importance of studying regional inequality at different spatial levels, as the necessary precondition to formulate pragmatic and effective policies. The results indicate that FDI is negatively associated with intra-provincial inequality, whereas international trade does not seem to have a significant effect. The COVID-19 has significantly affected the FDI and globalization, most of the countries were shut down the border and this would enlarge the inequality in China. However, the FDI would increase after COVID-19 and we proposes the following policy implications. First, FDI should be encouraged by the government, especially in poor regions. Second, more preferential administrative policies, tax incentives, and improved transportation infrastructure should be provided to the underdeveloped regions, to enable easier access and to attract more FDI.

We also document that intra-provincial inequality increases as the primary industry sector (agriculture) declines. This suggests that the Chinese government can reduce regional inequality by stimulating growth in the primary industry sector as suggested by e.g., Ravallion and Chen (32) and Chen and Groenewold (57). However, it would be inappropriate to restrict the development of the secondary and tertiary industry sectors in redressing inequality because the development in these sectors can greatly reduce the poverty at the aggregate level. Moreover, restricting the development of the above sectors can make the population in the poor regions worse-off, even if intra-provincial inequality is reduced. In other words, the increase in inequality stems not from the development in the tertiary or secondary industry sectors per se, but the unevenness in the distribution of these sectors. Therefore, the policymakers should not abandon globalization, industrialization, and development in secondary and tertiary industries, but instead, direct them toward disadvantaged regions and ensure that they benefit the poor, i.e., spread far into the poor regions within a particular province.

## Data Availability Statement

The original contributions presented in the study are included in the article/supplementary material, further inquiries can be directed to the corresponding author.

## Author Contributions

TC: conceptualization, data curation, methodology, visualization, writing—original draft preparation, and writing—review and editing. YW: conceptualization, data curation, methodology, software, visualization, writing—original draft preparation, and writing—review and editing. MW: data curation, methodology, software, visualization, writing—original draft preparation, and writing—review and editing. NM: data curation, methodology, visualization, writing—original draft preparation, and writing—review and editing. All authors contributed to the article and approved the submitted version.

## Funding

This work was supported by the Hainan College of Economics and Business (Project Reference Number: hnjmk2021301).

## Conflict of Interest

The authors declare that the research was conducted in the absence of any commercial or financial relationships that could be construed as a potential conflict of interest.

## Publisher's Note

All claims expressed in this article are solely those of the authors and do not necessarily represent those of their affiliated organizations, or those of the publisher, the editors and the reviewers. Any product that may be evaluated in this article, or claim that may be made by its manufacturer, is not guaranteed or endorsed by the publisher.

## Footnotes

^1^According to the National Bureau of Statistics of China (thereafter NBSC).

^2^The word ‘globalization' could mean different things. In this study we refer to economic globalization measured by the scope of foreign direct investment (FDI) and international trade (imports and exports).

^3^The noticeable exceptions are studies based on prefectural-level regional data conducted by Wei and Wu (44), Huang and Wei (19), and Zhang et al. (20) who examined income inequalities amongst prefectural-level Chinese cities.

^4^For the list of all the variables and their definitions see Table A1 in the Appendix.

^5^We exclude four municipalities: Beijing, Tianjin, Shanghai and Chongqing because the majority of their administrative regions are districts, not counties or county-level cities.

^6^China's GDP composes of three broad sectors: primary industry (mainly agriculture), secondary industry (manufacturing and construction) and tertiary industry (services).

^7^The regional inequality indicator (*GINI*) used in this study refers to the inequality amongst the cities and counties within a province excluding the districts. Therefore, for the sake of consistency, the *GRPPC* factor for each province is also based on data from all the cities and counties in that province, while excluding the districts.

^8^To handle the problem of unequal capacities (volumes of transport per mile) in three major modes of transportation, i.e., highways, railways, and waterways, we follow the approach used by Yao and Wei (59). That is, we convert railways (waterways) into equivalent highways using the conversion ratio of 4.27 (1.06).

^9^All the data series were tested for the presence of unit root using the Fisher ADF and Fisher PP test. Most of the data series fail both tests which constitutes another argument for using 2S-SGMM estimator.

^10^Untabulated estimated results from ordinary least squares (OLS), fixed effects (FE), random effects (RE), and two-way fixed effects are used as a robustness measure. Most of the untabulated estimated coefficients have similar statistical significance and signs to those in Table 2. Therefore, our estimated results are fairly robust.

^11^Following e.g., Firth et al. (66), this result is calculated as follows: 0.013^*^0.1^*^-3.526/0.283.

## Appendix

**Table A1:** Variables used in the baseline model (presented in Table 2).

**Name**	**Definition**
**Dependent variable**	
*GINI*	Intra-provincial regional inequality for each province
**Explanatory variables**	
*FDI*	Foreign Direct Investment/provincial GRP
*XIM*	The total value of exports and imports/provincial GRP
*DOMTR*	Retail sales of consumer goods/provincial GRP
*SECIND*	Secondary industry sector GRP/provincial GRP
*TERIND*	Tertiary industry sector GRP/provincial GRP
*GRPPC*	Real GRP per capita (1,000 Yuan)
*TRANSP*	Transportation infrastructure (1,000 km)
*INLF*	The provincial CPI (price base of the previous year is treated as 100)
*GOVEX*	Government expenditure for supporting underdeveloped areas/provincial GRP
*ECOSIG*	Provincial GRP/national GDP
*EDUFND*	Educational funding/provincial GRP
